# Diagnostic Performance of Two Point-of-Care Tests for Anti-HCV Detection

**DOI:** 10.5812/hepatmon.12274

**Published:** 2013-09-10

**Authors:** Lígia da Rosa, Esther Buzaglo Dantas-Corrêa, Janaína Luz Narciso-Schiavon, Leonardo de Lucca Schiavon

**Affiliations:** 1 Division of Gastroenterology, Federal University of Santa Catarina, Santa Catarina, Brazil

**Keywords:** Point-of-Care Systems, Hepatitis C, Diagnosis

## Abstract

**Background:**

Besides the great importance of the issue in terms of public health, there is a lack of studies evaluating the performance of several of the currently used point of care tests (POCTs) for the detection of anti-HCV.

**Objectives:**

To investigate the performance of two POCTs for anti-HCV detection and to assess the impact of the reading time on diagnostic performance.

**Patients and Methods:**

A total of 307 subjects were divided into three groups (1- HCV infected; 2- other chronic liver diseases; and 3- controls). The POCTs HCV Rapid Test Bioeasy® and Imuno-Rapido HCV® were read at 3, 5, 10, 15, 20 and 30 minutes. The sensitivity and specificity of the POCTs were calculated in relation to anti-HCV detection by chemiluminescence.

**Results:**

Valid results were obtained for all tests. When compared to the chemiluminescence, both tests showed sensitivity of 97.1% and specificity of 100%. No changes in the sensitivity or specificity of the tests were observed at different reading times and when patients with other chronic liver diseases were evaluated as a control group.

**Conclusions:**

The POCTs evaluated in this study showed high sensitivity and specificity, with no change in the performance after the third minute of reading.

## 1. Background

Over the last few years, hepatitis C virus (HCV) infection has emerged as one of the most significant causes of chronic liver disease worldwide, with estimated prevalence ranging from 2.2 to 3.0% (1). Additionally, a significant proportion of HCV infected subjects will ultimately evolve to liver cirrhosis and/or hepatocellular carcinoma, making chronic HCV infection a major health problem (2, 3). Despite the excellent accuracy of the currently available tests for the detection of anti-HCV antibodies, the delay in reporting the results, the need for specialized equipment for processing the samples and interpreting the results, as well as the need to transfer individuals to sample collection and processing centers, limit their use as screening tools. Serologic point of care tests (POCTs) have several advantages, namely that they require little specialized apparatus, can be brought to the individuals who are to be tested and allow diagnosis in as little as a few minutes in different clinical settings (4). These advantages might be translated into increased testing opportunity and, ultimately, identification of more patients who could benefit from antiviral treatment (5). Over the last few years, several tests for rapid detection of anti-HCV have been developed and are currently in use in various countries; however, only recently the first POCT was approved by the U.S. Food and Drug Administration (6). The investigation of the diagnostic accuracy of POCTs and rapid tests for the detection of anti-HCV is a highly relevant topic. Besides the great importance of the issue in terms of public health, there is a lack of studies evaluating the performance of several of the currently used tests.

## 2. Objectives

Our goals were to investigate the performance of two immunochromatographic POCTs for the detection of anti-HCV antibodies and to assess the impact of the reading time on diagnostic performance.

## 3. Patients and Methods

This is a cross-sectional study that was performed in an outpatient clinic of a hepatology reference service located in Florianopolis, Brazil, in the period between January 2010 and May 2011. The individuals were divided into three groups: 1) Case group = patients with chronic HCV (individuals known to have chronic HCV, as diagnosed by PCR for HCV-RNA); 2) Control group 1 = patients with other chronic liver diseases (non-HCV carriers); and 3) Control group 2 = subjects without chronic liver disease (individuals with non-reagent anti-HCV and without a clinical history of chronic liver disease). The Control group 2 individuals were blood donors, hospital staff not involved in invasive procedures or individuals from cardiology outpatient clinic. This group was age and gender-matched to the HCV infected group. Immunosuppressed individuals (patients undergoing chemotherapy or immunosuppressive treatment or who were co-infected with human immunodeficiency virus); individuals undergoing treatment with interferon and patients with chronic kidney disease and on hemodialysis were excluded. All subjects from the three groups were HIV negative. The minimum sample size for each group was estimated at 95 individuals, considering a maximum margin of error of 2%, a 95% confidence interval and estimated diagnostic sensitivity and specificity of 99%. The final sample size comprises 307 individuals (103 in Case group, 101 in Control group 1 and 103 in Control group 2). Informed consent was obtained from each patient included in the study and the study protocol conforms to the ethical guidelines of the 1975 Declaration of Helsinki as reflected in a priori approval by the Federal University of Santa Catarina human research committee.

The individuals were evaluated for inclusion during routine outpatient visits. After the patients were informed of the study procedures and provided informed consent, the rapid test was performed using digital puncture (10 µL of whole blood, according to the manufacturer’s instructions). Immediately following the completion of the rapid test, a sample collection by peripheral venipuncture for an amplified chemiluminescence test for anti-HCV (Architect system, Abbott Diagnostics Division, Illinois, USA) was performed in all individuals. The POCTs studied were the HCV Rapid Test Bioeasy® (Standard Diagnostics, Yongin, Korea) and Imuno-Rapido HCV® (Wama Diagnostica, São Carlos, Brazil). Both tests rely on the immunochromatographic method using synthetic and recombinant antigens (Core, NS3, NS4, NS5). All test protocols were carried out by the same examiner strictly according to the guidelines of the manufacturers. The reading times suggested in the insert package were between 15 and 20 minutes for the HCV Rapid Test Bioeasy® and between 10 and 15 minutes for the Imuno-Rapido HCV®. Both manufacturers stated that the tests should not be interpreted after 20 minutes. In this experiment, tests were read at 3, 5, 10, 15, 20 and 30 minutes to investigate any changes in its performance. HCV-RNA was not performed for the purpose of this study; however, all patients in the case group were HCV-RNA positive. The polymerase chain reaction (PCR) method routinely adopted was the AMPLICOR® HCV Test 2.0 (Roche Molecular Systems, Branchburg, NJ, USA) with a lower detection limit of 50 IU/mL.

The Kolmogorov-Smirnov test was used to evaluate the normality of the distribution of variables. The continuous variables were compared using Student’s t-tests for normally distributed data or the Mann-Whitney test for non-normal distributions. The categorical variables were evaluated by the chi-square test. A P value of < 0.05 was considered statistically significant. The tests used were two-tailed and were performed by SPSS version 15.0 (SPSS, Chicago, IL, USA). The sensitivity and specificity of the POCTs were calculated in relation to anti-HCV detection by chemiluminescence (considered the gold standard in this study).

## 4. Results

The clinical and demographic variables of the three groups are shown in Table 1. When the Case group was evaluated regarding the major risk factors for infection, 32 subjects (31.1%) had received a blood transfusion (prior to 1992), and 23 (22.3%) reported having used intravenous drugs. The group of patients with HCV showed a lower proportion of Caucasians and higher ALT levels than the other groups and a higher mean age than the patients with liver diseases not related to HCV. Genotype was available for 56 HCV-infected subjects (54%), genotype 1 was observed in 34 and genotype 3 in 22 individuals.

The POCTs and chemiluminescence results are shown in Table 2. Valid results were obtained for all tests. When compared to the chemiluminescence, both the HCV Rapid Test Bioeasy® and Imuno-Rapido HCV® showed sensitivity of 97.1% (CI95%: 91,7% – 99,4%) and specificity of 100% (CI95%: 96,4% – 100%). No changes in the sensitivity or specificity of the tests were observed at different reading times neither when patients with other chronic liver diseases were evaluated as a control group. False-negative results for both brands of POCTs were observed in the same three individuals with confirmed HCV infection, two men and a woman aged between 45 and 54 years. The ALT levels were above the reference value in two of the false-negative result cases, and none of these patients had signs of advanced liver disease or an apparent cause of immunosuppression. No false-positive results were observed.

**Table 1.** Characteristics of the Individuals Included in the Study and Comparison of the Variables in Each Group

Variable	Case Group ^a^(n = 103)	Control Group 1 ^a^ (n = 101)	P value ^b^	Control Group 2 ^a^ (n = 103)	P value ^c^
**Age, y**					
Mean ± SD	54.66 ± 10.76	44.84 ± 13.54	< 0.001 ^e^	54.36 ± 11.15	0.884 ^e^
Median	54	46		54	
**Male gender, No. (%)**	56 (54.4)	44 (46.6)	0.123	56 (54.4)	1.000
**Caucasians, No. (%)**	79 (76.7)	90 (89.1)	0.019	97 (94.2)	< 0.001
**BMI ** ^**d**^ **(kg/m** ^**2**^ **)**					0.155 ^e^
Mean ± SD	26.63 ± 4.92	27.52 ± 6,00	0.260 ^f^	27.58 ± 4.28	
Median	26.37	26.43		27.33	
**ALT ** ^**d**^ **(IU/L) **					
Mean ± SD	90.87 ± 53.11	73.61 ± 55.58	< 0.001^f^	46.83 ± 18.37	< 0.001 ^f^
Median	79.50	56.00		42.00	

^a^ Case group = patients with chronic HCV; Control group 1 = patients with other chronic liver diseases (non-HCV carriers); Control group 2 = subjects without chronic liver disease (individuals with non-reagent anti-HCV and without a clinical history of chronic liver disease)

^b^ P-value for comparison between case group and control group 1

^c^ P-value for comparison between case group and control group 2

^d^ Abbreviations: BMI, Body mass index; ALT, Alanine aminotransferase

^e^ Student´s t test

^f^ Mann-Whitney U test

**Table 2.** Results of Anti-HCV Antibody Testing by the Point of Care Tests and Amplified Chemiluminescence

	HCV Rapid Test Bioeasy® ^a^		Imuno-Rapido HCV® ^a^		Anti-HCV Amplified Chemiluminescence	
	Positive	Negative	Positive	Negative	Positive	Negative
**Case group ** ^**a**^ **(n=103)**	100	3	100	3	103	0
**Control group 1 ** ^**b **^ **(n = 101)**	0	101	0	101	0	101
**Control group 2 ** ^**b **^ **(n = 103)**	0	103	0	103	0	103

^a^ Same results for all times of reading (3, 5, 10, 15, 20 and 30 minutes).

^b^ Case group = patients with chronic HCV; Control group 1 = patients with other chronic liver diseases (non-HCV carriers); Control group 2 = subjects without chronic liver disease (individuals with non-reagent anti-HCV and without a clinical history of chronic liver disease)

## 5. Discussion

In recent years, advances in detection technology have made a range of POCTs for different infectious diseases available. It is now possible to screen and diagnose those conditions at primary healthcare settings, using minimally invasive tests. In the present study, two not FDA-approved POCTs were performed in whole blood samples. The choice of the specimen was based on the fact that collection of plasma or serum samples requires equipment and training, and is more time consuming. Although the use of oral fluid is an attractive alternative, it is not recommended by the manufacturers of both tests performed here.

When compared to the chemiluminescence, both POCTs studied showed sensitivity of 97.1% (CI95%: 91.7% – 99.4%) and specificity of 100% (CI95%: 96.4% – 100%). Several POCTs for anti-HCV detection were previously evaluated with different performances. The FDA-approved OraQuick HCV Rapid Antibody Test (OraSure Technologies, Bethlehem, Pennsylvania) is one of the most studied rapid tests for the diagnosis of HCV infection. When using whole blood, this test exhibited sensitivity between 92.2% and 100%, and specificity between 97.2% and 100%, which is similar to our findings (7-9). Other whole blood POCTs, such as Anti-HCV Ab rapid test (Tema Ricerca, Bologna, Italy), SM-HCV Rapid Test (SEROMed Labor Spezialitaten, Pollenfeld, Germany), Multiplo Rapid HIV/HCV Antibody Test (MedMira, Halifax, Nova Scotia, Canada) were previously studied, with sensitivity and specificity ranging from 78.9% – 100% and 83.3% – 100%, respectively (9-11).

A recent meta-analysis that examined the diagnostic performance of POCTs and rapid tests for the diagnosis of hepatitis C showed that, although these tests generally have excellent specificity, they had a significant variation in sensitivity (22%-100%) (12). When the POC tests using whole blood were evaluated, the grouped sensitivity was 98.9%, and the grouped specificity was 99.5%. These results are similar to those observed in the present study. According to the manufacturer’s package insert information for the HCV Rapid Test Bioeasy® and Imuno-Rapido HCV®, in preliminary studies these tests exhibited sensitivities of 99% and 100% and specificities of 98.6% and 99.8%, respectively. However, to the best of our knowledge, this is the first independent study carried out with the imuno-Rapido HCV®. Regarding the Bioeasy test, one previous study evaluated its performance in an epidemiological survey of a high endemic Brazilian city (13). Although the POCT exhibited high sensitivity and specificity, this study is limited by the small number of individuals who were included in the test performance evaluation (30 cases and 41 controls).

There are several factors that could affect rapid anti-HCV tests accuracy. Immunosuppression, particularly HIV co-infection might be related to higher false-negative rates when employing HCV serological tests (12). In the present study, HIV infection and immunosuppression were exclusion criteria and additional studies are advisable to investigate the performance of the two POCTs employed here, in high risk populations for HIV infection. The influence of HCV genotype on rapid tests performance has been suggested (12, 14); however this is still a matter of discussion as genotypes are not reported in the majority of the studies. In this study, HCV genotype was available for only 54% individuals and the methodology was not intended to investigate this issue. Therefore, a genotype impact on the POCTs employed here cannot be ruled out.

No change in the performance of the two POCTs was observed after the third minute of reading in the present study. Although we cannot suggest a modification in the procedures for interpretation of tests results, these findings may be used as parameter for future studies aimed at evaluate POCTs for anti-HCV detection.

It is possible to conclude that the immunochromatographic POCTs used here (HCV Rapid Test Bioeasy® and imuno-Rapido HCV®) for the detection of anti-HCV showed high sensitivity and specificity and no change in the performance after the third minute of reading. Although future screening studies are required to confirm these data, these findings suggest that the tests results may be released more quickly than previously recommended, which could increase the adherence to hepatitis C screening campaigns.

## References

[1] Lavanchy D (2009). The global burden of hepatitis C.. Liver Int..

[2] Hoofnagle JH (1997). Hepatitis C: the clinical spectrum of disease.. Hepatology..

[3] Hutin Y, Kitler M, Dore G, Perz J, Armstrong G, Dusheiko G (2004). Global burden of disease (GBD) for hepatitis C.. J Clin Pharmacol..

[4] Ferreira-Gonzalez A, Shiffman ML Use of diagnostic testing for managing hepatitis C virus infection.. Semin liver dis..

[5] Tucker JD, Bien CH, Peeling RW (2013). Point-of-care testing for sexually transmitted infections: recent advances and implications for disease control.. Curr Opin Infect Dis..

[6] OraQuick HCV Rapid Antibody Test - P080027/S001.. http://www.fda.gov/MedicalDevices/ProductsandMedicalProcedures/DeviceApprovalsandClearances/Recently-ApprovedDevices/ucm246401.htm.

[7] Lee SR, Kardos KW, Schiff E, Berne CA, Mounzer K, Banks AT (2011). Evaluation of a new, rapid test for detecting HCV infection, suitable for use with blood or oral fluid.. J Virol Methods..

[8] Lee SR, Yearwood GD, Guillon GB, Kurtz LA, Fischl M, Friel T (2010). Evaluation of a rapid, point-of-care test device for the diagnosis of hepatitis C infection.. J Clin Virol..

[9] Smith BD, Teshale E, Jewett A, Weinbaum CM, Neaigus A, Hagan H (2011). Performance of Premarket Rapid Hepatitis C Virus Antibody Assays in 4 National Human Immunodeficiency Virus Behavioral Surveillance System Sites.. Clin Infect Dis..

[10] Hui AY, Chan FK, Chan PK, Tam JS, Sung JJ (2002). Evaluation of anew rapid whole-blood serological test for hepatitis c virus.. Acta Virol..

[11] Montebugnoli L, Borea G, Miniero R, Sprovieri G (1999). A rapid test for the visual detection of anti-hepatitis C virus antibodies in whole blood.. Clin Chim Acta..

[12] Shivkumar S, Peeling R, Jafari Y, Joseph L, Pant Pai N (2012). Accuracy of Rapid and Point-of-Care Screening Tests for Hepatitis CA Systematic Review and Meta-analysis.. Ann Intern Med..

[13] Ivantes CAP, Silva D, Messias-Reason I (2010). High prevalence of hepatitis C associated with familial history of hepatitis in a small town of south Brazil: efficiency of the rapid test for epidemiological survey.. Braz J Infect Dis..

[14] Pai NP, Sollis K, Peeling RW (2013). Rapid hepatitis C tests: better than the gold standard?. Expert Rev Mol Diagn..

